# Supplementary material for: Impact of explicit area scaling on kinetic models involving multiple compartments
Source: BMC Bioinformatics. 2021 Jan 11;22:21. doi: 10.1186/s12859-020-03913-8 (PMC7798250; doi:10.1186/s12859-020-03913-8)
Supplement: Supplementary file 1 — Additional file 1: List of curated, multi-compartment models in the biomodels database with realistic compartment sizes (Table S1). Detailed description of the models used in this study, including the setup and parameters of the transport model (Tables S2, S3), the changes to the RanGTP transport and the TGF-beta signaling model as well as the calculated scaled sensitivity values for both models (Tables S4, S5). [file 12859_2020_3913_MOESM1_ESM.pdf]

1 Models with realistic compartment sizes from the BioModels databse

Table 1 Curated multi-compartment models in the biomodels database. Listed are only those models with realistic compartment sizes with biomodel number (#) [1] (https://wwwdev.ebi.ac.uk/biomodels/, last accessed: 14.11.2019). The chosen example models are highlighted in bold font. Models built in Virtual Cell [2] are marked by VC.

#	Description	Reference
16	Circadian oscillations in the *drosophila* period protein (PER)	[3]
19	Dynamics of the MAP kinase cascade activated by surface and internalized EGF receptors	[4]
59	Ca^{2+} flux in pancreatic beta-cells: role of the plasma membrane and intracellular stores	[5]
75 (VC)	Kinetic analysis of receptor-activated phosphoinositide turnover	[6]
122, 123	NFAT and NFκB activation in T lymphocytes	[7]
161 (VC)	Compartment-specific feedback loop and regulated trafficking can result in sustained activation of Ras at the Golgi	[8]
162 (VC)	Modeling and analysis of calcium signaling events leading to long-term depression in cerebellar Purkinje cells.	[9]
173	Smad nucleocytoplasmic shuttling as a dynamic signal-interpreting system	[10]
182 (VC)	Cell shape and negative links in regulatory motifs together control spatial information flow in signaling networks.	[11]
**192**	Ran-driven cargo transport and the RanGTPase system	[12]
250	Ligand-specific c-Fos expression	[13]
**342**	Transient and sustained transforming growth factor-β signaling	[14]
399	Thermodynamically consistent model calibration in chemical kinetics	[15]
407	Heterogeneity reduces sensitivity of cell death for TNF-stimuli	[16]
474	Regulation of insulin signaling by oxidative stress	[17]
482	The selective control of glycolysis, gluconeogenesis and glycogenesis by temporal insulin patterns	[18]
505, 506	Fatty acid β-oxidation	[19]
547	Arsenic transport, distribution and detoxification in yeast	[20]
610	Quantitavie analysis of glycerol accumulation, glycolysis and growth under hyper osmotic stress	[21]
638	Yeast GPCR signaling reflects the fraction of occupied receptors	[22]
699, 702	Regulation of Tem1 by the GAP complex in Spindle Position Checkpoint	[23]
705, 706	Response of FOXO transcription factors to multiple post-translational modifications made by ageing-related signaling pathways	[24]

2 Setup of the transport model

The model comprises only one reversible reaction:

$X_1 \leftrightarrow X_2$

In the following equations, concentrations are noted as $[X]$, V refers to cellular volumes, $A_{interaction}$ defines the interaction area between cells and $k_{1,2,A1,A2}$ are kinetic parameters.

One compartment

$$-\frac{[X_1] \cdot V_{cell}}{dt} = \frac{[X_2] \cdot V_{cell}}{dt} = V_{cell} * (k_1 * [X_1] - k_2 * [X_2]) \quad (1)$$

Volume scaling transport

$$-\frac{[X_1] \cdot V_{cell\,1}}{dt} = \frac{[X_2] \cdot V_{cell\,2}}{dt} = V_{cell\,1} * k_1 * [X_1] - V_{cell\,2} * k_2 * [X_2] \quad (2)$$

Area scaling transport

$$-\frac{[X_1] \cdot V_{cell\,1}}{dt} = \frac{[X_2] \cdot V_{cell\,2}}{dt} = A_{interaction} * (k_{A1} * [X_1] - k_{A2} * [X_2]) \quad (3)$$

3 Parameters of the transport model

Table 2 Parameters of the transport model for the first transition (transition to meristematic zone) and all three modeling approaches.

model structure	model entity	value
one compartment	k_1	$0.1\,s^{-1}$
	k_2	$0.1\,s^{-1}$
volume scaling	k_1	$0.1\,s^{-1}$
	k_2	$0.1\,s^{-1}$
area scaling	k_{A1}	$8.8192 * 10^{-6}\,dm * s^{-1}$
	k_{A2}	$8.8192 * 10^{-6}\,dm * s^{-1}$

Table 3 Compartment sizes of the different transitions for the toy model. For the one compartment model, the cell has the volume listed in the column cell 1. The interaction area was only used for the area-scaling model.

transition	cell 1	cell 2	interaction area
1	$8.47 * 10^{-13}\,l$	$8.47 * 10^{-13}\,l$	$9.604 * 10^{-9}\,dm^2$
2	$2.271 * 10^{-12}\,l$	$8.47 * 10^{-13}\,l$	$1.69 * 10^{-8}\,dm^2$
3	$5.803 * 10^{-12}\,l$	$2.271 * 10^{-12}\,l$	$2.1904 * 10^{-8}\,dm^2$
4	$1.6364 * 10^{-11}\,l$	$5.803 * 10^{-12}\,l$	$2.5281 * 10^{-8}\,dm^2$

4 RanGTP transport model

4.1 Altered reactions

Cytoplasmic transfer $(RanGTP_{nuc} \leftrightarrow RanGTP_{cy})$

new reaction rate law:

$k_{A\,perm\,RanGTP} * A_{nucleus} * (RanGTP_{nuc} - RanGTP_{cy})$

with $k_{A\,perm\,RanGTP}$: $1.429 * 10^{-6}\,dm * s^{-1}$

$Nucleoplasmic\ transfer \quad (RanGDP_{nuc} \leftrightarrow RanGDP_{cy})$
new reaction rate law:
$k_{A\,perm\,RanGDP} * A_{nucleus} * (RanGDP_{nuc} - RanGDP_{cy})$
with $k_{A\,perm\,RanGDP}$: $5.714 * 10^{-6}\, dm * s^{-1}$

4.2 Sensitivities

Table 4 Scaled sensitivities of the nuclear RanGTP concentration with respect to model parameters at steady state in the original, volume-scaling model by Görlich *et al.*.

reaction	parameter	scaled sensitivity
RCC1_binding	k_1	0.139263
RCC1_binding	k_2	-0.13091
GDP_dissociation	k_1	0.133022
GDP_dissociation	k_2	-0.110622
GTP_binding	k_1	0.112722
GTP_binding	k_2	-0.111463
RanGTP_release	k_1	0.145492
RanGTP_release	k_2	-0.144969
Cytoplasmic_transfer	$k_{A\,perm\,RanGTP}$	-0.257474
Nucleoplasmic_transfer	$k_{A\,perm\,RanGDP}$	0.223293
RanGTP_binding	k_1	$5.06681 * 10^{-05}$
RanGTP_binding	k_2	$-2.68329 * 10^{-10}$
RanBP1_RanGDP	K_m	$-1.2809 * 10^{-05}$
RanBP1_RanGDP	k_{cat}	$1.2804 * 10^{-05}$
RanGAP_RanGDP	$K_{m\,GAP}$	-0.00109736
RanGAP_RanGDP	$k_{cat\,GAP}$	0.00110842

5 Smad2 signalling model

5.1 altered reactions

$R1\_Smad2\_import \quad (Smad2\_c \rightarrow Smad2\_n)$
new reaction rate law:
$A_{nucleus} * k_{A\,imp}\_Smad2c * Smad2c$
with $k_{A\,imp}\_Smad2c$: $7 * 10^{-3}\, dm * s^{-1}$

$R2\_Smad2\_export \quad (Smad2\_n \rightarrow Smad2\_c)$
new reaction rate law:
$A_{nucleus} * k_{A\,exp}\_Smad2n * Smad2n$
with $k_{A\,exp}\_Smad2n$: $1.4583 * 10^{-2}\, dm * s^{-1}$

$R3\_Smad4\_import \quad (Smad4\_c \rightarrow Smad4\_n)$
new reaction rate law:
$A_{nucleus} * k_{A\,imp}\_Smad4c * Smad4c$
with $k_{A\,imp}\_Smad4c$: $3.5 * 10^{-3}\, dm * s^{-1}$

$R4\_Smad4\_export \quad (Smad4\_n \rightarrow Smad4\_c)$
new reaction rate law:
$A_{nucleus} * k_{A\,exp}\_Smad4c * Smad4c$
with $k_{A\,exp}\_Smad4c$: $7.2917 * 10^{-3}\,dm * s^{-1}$

$R24\_Smads\_Complex\_import \quad (Smads\_Complex\_c \rightarrow Smads\_Complex\_n)$
new reaction rate law:
$A_{nucleus} * k_{A\,imp}\_Smads\_Complex\_c * Smads\_Complex\_c$
with $k_{A\,imp}\_Smads\_Complex\_c$: $7 * 10^{-3}\,dm * s^{-1}$

5.2 Sensitivities

Table 5 Scaled sensitivities of the maximal nuclear Smads_Complex concentration with respect to model parameters in the original, volume-scaling model.

reaction	parameter	scaled sensitivity
R1_Smad2_import	$k_{A\,imp}\_Smad2c$	-0.0666527
R2_Smad2_export	$k_{A\,exp}\_Smad2n$	0.0916061
R3_Smad4_import	$k_{A\,imp}\_Smad4c$	-0.0666877
R4_Smad4_export	$k_{A\,exp}\_Smad4n$	0.0819686
R5_T1R_production	v_{T1R}	0.024528
R6/R12/R18_T1R/T2R/LRC_Cave_formation	ki_{Cave}	-0.340296
R7/R13/R19_T1R/T2R/LRC_Cave_recycling	kr_{Cave}	0.0842739
R8/R14/R20_T1R/T2R/LRC_EE_formation	ki_{EE}	0.302112
R9/R15/R21_T1R/T2R/LRC_EE_recycling	kr_{EE}	-0.130795
R10_T1R_EE_degradation	$k_{deg}\_T1R\_EE$	-0.00708498
R11_T2R_production	v_{T2R}	0.129776
R16_T2R_EE_degradation	$k_{deg}\_T2R\_EE$	-0.0224511
R17_LRC_formation	k_{LRC}	0.0242155
R22_LRC_EE_degradation	k_{cd}	-0.0550185
R23_Smads_Complex_formation	$k\_Smads\_Complex\_c$	0.486002
R24_Smads_Complex_import	$k_{A\,imp}\_Smads\_Complex_c$	0.198014
R25_Smads_Complex_Dissociation	$k_{diss}\_Smads\_Complex\_n$	-0.722791
R26_LRC_Cave_degradation	k_{lid}	-0.00316132

Author details

References
1. Li, C., Donizelli, M., Rodriguez, N., Dharuri, H., Endler, L., Chelliah, V., Li, L., He, E., Henry, A., Stefan, M.I., Snoep, J.L., Hucka, M., Le Novère, N., Laibe, C.: BioModels Database: An enhanced, curated and annotated resource for published quantitative kinetic models. BMC Systems Biology **4**(1), 92 (2010). doi:10.1186/1752-0509-4-92
2. Schaff, J., Fink, C.C., Slepchenko, B., Carson, J.H., Loew, L.M.: A general computational framework for modeling cellular structure and function. Biophysical Journal **73**(3), 1135–1146 (1997). doi:10.1016/S0006-3495(97)78146-3
3. Goldbeter, A.: A model for circadian oscillations in the Drosophila period protein (PER). Proceedings. Biological sciences **261**(1362), 319–24 (1995). doi:10.1098/rspb.1995.0153
4. Schoeberl, B., Eichler-Jonsson, C., Gilles, E.D., Müller, G.: Computational modeling of the dynamics of the MAP kinase cascade activated by surface and internalized EGF receptors. Nature biotechnology **20**(4), 370–5 (2002). doi:10.1038/nbt0402-370
5. Fridlyand, L.E., Tamarina, N., Philipson, L.H.: Modeling of Ca2+ flux in pancreatic beta-cells: role of the plasma membrane and intracellular stores. American journal of physiology. Endocrinology and metabolism **285**(1), 138–54 (2003). doi:10.1152/ajpendo.00194.2002

6. Xu, C., Watras, J., Loew, L.M.: Kinetic analysis of receptor-activated phosphoinositide turnover. The Journal of cell biology **161**(4), 779–91 (2003). doi:10.1083/jcb.200301070
7. Fisher, W.G., Yang, P.-C., Medikonduri, R.K., Jafri, M.S.: NFAT and NFkappaB activation in T lymphocytes: a model of differential activation of gene expression. Annals of biomedical engineering **34**(11), 1712–28 (2006). doi:10.1007/s10439-006-9179-4
8. Eungdamrong, N.J., Iyengar, R.: Compartment-specific feedback loop and regulated trafficking can result in sustained activation of Ras at the Golgi. Biophysical journal **92**(3), 808–15 (2007). doi:10.1529/biophysj.106.093104
9. Hernjak, N., Slepchenko, B.M., Fernald, K., Fink, C.C., Fortin, D., Moraru, I.I., Watras, J., Loew, L.M.: Modeling and analysis of calcium signaling events leading to long-term depression in cerebellar Purkinje cells. Biophysical journal **89**(6), 3790–806 (2005). doi:10.1529/biophysj.105.065771
10. Schmierer, B., Tournier, A.L., Bates, P.A., Hill, C.S.: Mathematical modeling identifies Smad nucleocytoplasmic shuttling as a dynamic signal-interpreting system. Proceedings of the National Academy of Sciences of the United States of America **105**(18), 6608–13 (2008). doi:10.1073/pnas.0710134105
11. Neves, S.R., Tsokas, P., Sarkar, A., Grace, E.A., Rangamani, P., Taubenfeld, S.M., Alberini, C.M., Schaff, J.C., Blitzer, R.D., Moraru, I.I., Iyengar, R.: Cell shape and negative links in regulatory motifs together control spatial information flow in signaling networks. Cell **133**(4), 666–80 (2008). doi:10.1016/j.cell.2008.04.025
12. Görlich, D., Seewald, M.J., Ribbeck, K.: Characterization of Ran-driven cargo transport and the RanGTPase system by kinetic measurements and computer simulation. The EMBO journal **22**(5), 1088–100 (2003). doi:10.1093/emboj/cdg113
13. Nakakuki, T., Birtwistle, M.R., Saeki, Y., Yumoto, N., Ide, K., Nagashima, T., Brusch, L., Ogunnaike, B.A., Okada-Hatakeyama, M., Kholodenko, B.N.: Ligand-specific c-Fos expression emerges from the spatiotemporal control of ErbB network dynamics. Cell **141**(5), 884–96 (2010). doi:10.1016/j.cell.2010.03.054
14. Zi, Z., Feng, Z., Chapnick, D.A., Dahl, M., Deng, D., Klipp, E., Moustakas, A., Liu, X.: Quantitative analysis of transient and sustained transforming growth factor-β signaling dynamics. Molecular systems biology **7**(1), 492 (2011). doi:10.1038/msb.2011.22
15. Jenkinson, G., Goutsias, J.: Thermodynamically consistent model calibration in chemical kinetics. BMC systems biology **5**(1), 64 (2011). doi:10.1186/1752-0509-5-64
16. Schliemann, M., Bullinger, E., Borchers, S., Allgöwer, F., Findeisen, R., Scheurich, P.: Heterogeneity reduces sensitivity of cell death for TNF-stimuli. BMC systems biology **5**(1), 204 (2011). doi:10.1186/1752-0509-5-204
17. Smith, G.R., Shanley, D.P.: Computational modelling of the regulation of Insulin signalling by oxidative stress. BMC systems biology **7**(1), 41 (2013). doi:10.1186/1752-0509-7-41
18. Noguchi, R., Kubota, H., Yugi, K., Toyoshima, Y., Komori, Y., Soga, T., Kuroda, S.: The selective control of glycolysis, gluconeogenesis and glycogenesis by temporal insulin patterns. Molecular Systems Biology **9**(1), 664 (2013). doi:10.1038/msb.2013.19
19. van Eunen, K., Simons, S.M.J., Gerding, A., Bleeker, A., den Besten, G., Touw, C.M.L., Houten, S.M., Groen, B.K., Krab, K., Reijngoud, D.-J., Bakker, B.M.: Biochemical competition makes fatty-acid β-oxidation vulnerable to substrate overload. PLoS computational biology **9**(8), 1003186 (2013). doi:10.1371/journal.pcbi.1003186
20. Talemi, S.R., Jacobson, T., Garla, V., Navarrete, C., Wagner, A., Tamás, M.J., Schaber, J.: Mathematical modelling of arsenic transport, distribution and detoxification processes in yeast. Molecular microbiology **92**(6), 1343–56 (2014). doi:10.1111/mmi.12631
21. Petelenz-Kurdziel, E., Kuehn, C., Nordlander, B., Klein, D., Hong, K.K., Jacobson, T., Dahl, P., Schaber, J., Nielsen, J., Hohmann, S., Klipp, E.: Quantitative Analysis of Glycerol Accumulation, Glycolysis and Growth under Hyper Osmotic Stress. PLoS Computational Biology **9**(6), 1003084 (2013). doi:10.1371/journal.pcbi.1003084
22. Bush, A., Vasen, G., Constantinou, A., Dunayevich, P., Patop, I.L., Blaustein, M., Colman-Lerner, A.: Yeast GPCR signaling reflects the fraction of occupied receptors, not the number. Molecular systems biology **12**(12), 898 (2016)
23. Caydasi, A.K., Lohel, M., Grünert, G., Dittrich, P., Pereira, G., Ibrahim, B.: A dynamical model of the spindle position checkpoint. Molecular systems biology **8**, 582 (2012). doi:10.1038/msb.2012.15
24. Smith, G.R., Shanley, D.P.: Modelling the Response of FOXO Transcription Factors to Multiple Post-Translational Modifications Made by Ageing-Related Signalling Pathways. PLoS ONE **5**(6), 11092 (2010). doi:10.1371/journal.pone.0011092
